# Carbon Ion Radiotherapy Acts as the Optimal Treatment Strategy for Unresectable Liver Cancer During the Coronavirus Disease 2019 Crisis

**DOI:** 10.3389/fpubh.2021.767617

**Published:** 2021-12-09

**Authors:** Zheng Li, Qiang Li, Xiaohu Wang, Sha Li, Weiqiang Chen, Xiaodong Jin, Xinguo Liu, Zhongying Dai, Xiongxiong Liu, Xiaogang Zheng, Ping Li, Hui Zhang, Qiuning Zhang, Hongtao Luo, Ruifeng Liu

**Affiliations:** ^1^Institute of Modern Physics, Chinese Academy of Sciences (CAS), Lanzhou, China; ^2^Key Laboratory of Heavy Ion Radiation Biology and Medicine of Chinese Academy of Sciences, Lanzhou, China; ^3^Gansu Provincial Key Laboratory of Basic Research on Heavy Ion Radiation Application in Medicine, Lanzhou, China; ^4^Lanzhou Heavy Ion Hospital, Lanzhou, China; ^5^University of Chinese Academy of Sciences, Beijing, China; ^6^The 940th Hospital of Joint Logistics Support Force of Chinese People's Liberation Army, Lanzhou, China

**Keywords:** liver neoplasms, carbon ion radiotherapy, telemedicine, COVID-19, SARS-CoV-2, medical resource

## Abstract

The coronavirus disease 2019 (COVID-19) pandemic has greatly disrupted the normal treatment of patients with liver cancer and increased their risk of death. The weight of therapeutic safety was significantly amplified for decision-making to minimize the risk of severe acute respiratory syndrome coronavirus 2 (SARS-CoV-2) infection. Herein, the safety and effectiveness of carbon ion radiotherapy (CIRT) for unresectable liver cancer (ULC) were evaluated, and Chinese experiences were shared to solve the predicament of ULC treatment caused by SARS-CoV-2. Worldwide studies were collected to evaluate CIRT for ULC as the world has become a community due to the COVID-19 pandemic. We not only searched five international databases including the Cochrane Library, Web of Science, PubMed, Embase, and Scopus but also performed supplementary retrieval with other sources. Chinese experiences of fighting against COVID-19 were introduced based on the advancements of CIRT in China and a prospective clinical trial of CIRT for treating ULC. A total of 19 studies involving 813 patients with ULC were included in the systematic review. The qualitative synthetic evaluation showed that compared with transarterial chemoembolization (TACE), CIRT could achieve superior overall survival, local control, and relative hepatic protection. The systematic results indicated that non-invasive CIRT could significantly minimize harms to patients with ULC and concurrently obtain superior anti-cancer effectiveness. According to the Chinese experience, CIRT allows telemedicine within the hospital (TMIH) to keep a sufficient person-to-person physical distance in the whole process of treatment for ULC, which is significant for cutting off the transmission route of SARS-CoV-2. Additionally, CIRT could maximize the utilization rate of hospitalization and outpatient care (UHO). Collectively, CIRT for ULC patients not only allows TMIH and the maximized UHO but also has the compatible advantages of safety and effectiveness. Therefore, CIRT should be identified as the optimal strategy for treating appropriate ULC when we need to minimize the risk of SARS-CoV-2 infection and to improve the capacity of medical service in the context of the unprecedented COVID-19 crisis.

## Introduction

The coronavirus disease 2019 (COVID-19) pandemic, an infectious disease caused by a novel coronavirus named severe acute respiratory syndrome coronavirus 2 (SARS-CoV-2) (1, 2), was declared a pandemic by the WHO on March 11, 2020 (3). COVID-19 has been spreading around the world and bringing unprecedented catastrophe to humans (2–4). The figures released by WHO on November 23, 2021 showed that SARS-CoV-2 had infected more than 257.46 million people and caused more than 5.15 million deaths in over 220 countries and regions worldwide (4). The COVID-19 pandemic has impacted every aspect of human life, especially in the health care of all countries (5, 6). Patients with cancer are susceptible to being infected by it because of the poor systemic immunosuppressive state caused by the malignancy and conventional anticancer treatments, such as surgery or chemotherapy (7–12). Moreover, cancer and its conventional treatments are associated with deteriorating conditions and a worse prognosis of patients with COVID-19 concomitant (7–12). In order to reduce the risk of SARS-CoV-2 infection, postponing treatment was proposed in some guidelines to adjust cancer management (13); however, it is becoming increasingly inapplicable because of the increasing cancer malignant death (5, 14, 15). What is the solution for this dilemmatic predicament caused by the COVID-19 pandemic (16)? Obviously, we should find a way of fighting against cancer and SARS-CoV-2 synchronously (4, 14, 15). Some evidence has indicated that the optimization of anti-cancer safety is a realistic and feasible solution for the predicament during the COVID-19 crisis (7–12). What is the revised optimal treatment strategy for unresectable liver cancer (ULC) in the context of the COVID-19 crisis?

The weight of therapeutic safety is enlarged due to SARS-CoV-2 (7–12). Therefore, non-invasiveness and telemedicine within the hospital (TMIH) should be the crucial considerations for anti-cancer treatment during the COVID-19 crisis, especially for patients in the worst-hit areas (7–12). The principles of non-invasiveness and TMIH are necessary to get the optimal risk-benefit results in the fighting against SARS-CoV-2 and liver cancer synchronously (7–12, 17–19). There are unique superiorities of non-invasive carbon ion radiotherapy (CIRT) (20, 21), especially when it comes to the ability of TMIH concerning the controllable risk of SARS-CoV-2 infection, as well as preserving the patient's systemic function (including immunity) at relatively good levels to reduce the risk of SARS-CoV-2 infection (21–30). Several similar studies are helpful to fully understand the unique potentiality of CIRT in preserving cancer patients from the SARS-CoV-2 infection (31–35). Therefore, non-invasive CIRT seems to be the optimal strategy among multifarious therapies for treating ULC during the COVID-19 crisis when an oncologist needs to minimize the risk of SARS-CoV-2 infection (7–12, 21–30). However, the evidence for decision-making is lacking in terms of CIRT for ULC. Accordingly, the safety and effectiveness of CIRT for treating ULC were comprehensively evaluated by this systematic review to give evidence-based references in decision-makings and the advancements of CIRT in China together with clinical experiences were shared to provide references for other countries struggling with SARS-CoV-2 and liver cancer.

## Materials and Methods

A pre-retrieval procedure was implemented to ensure that the best results of literature retrieval could be obtained, which started on March 11, 2020. A preliminary and rapid systematic review was conducted before this study to ascertain how to design this study scientifically and accurately.

### Inclusion and Exclusion Criteria of Study Selection

Studies were included if they matched the following criteria based on the pilot study of a systematic review. (1) Participants: patients were diagnosed with liver cancer by histopathology and imageological examination, ineligible or infeasible for resection; regardless of primary liver cancer or metastatic liver cancer. (2) Intervention and comparison: there were few studies with a control group for the assessment of CIRT in treating patients with liver cancer on the basis of pre-retrieval. Therefore, a study should be included if CIRT was evaluated with effectiveness and/or adverse effects in treating liver cancer, whether there was a comparison group or not. (3) Outcomes: the outcomes of evaluation included overall survival (OS), local control, short-term effects, adverse effects, and complications. (4) The study type was unrestricted due to the development stage of CIRT. All study types of clinical research were included to evaluate CIRT for liver cancer on the basis of the pre-retrieval and preliminary systematic review. Publications were excluded if they had inappropriate research designs including cellular or animal experiments, letters, editorials, commentaries, protocols, reviews, systematic reviews, or meta-analyses.

### Search Strategy and Study Screening

The pre-retrieval was performed on March 11, 2020 and the comprehensive retrieval was started on April 15, 2020, following the pilot systematic review. The retrieval was updated every month during the research process in order to acquire the latest data of reports. The final retrieval time was May 31, 2021.

We searched five international databases including the Cochrane Library, Web of Science, PubMed, Embase, and Scopus from the database inception to May 31, 2021. We also searched other supplementary resources, such as Google Scholar, Medical Matrix, reference lists of relevant reviews and included papers, COVID-19 Open Research Dataset Challenge (CORD-19), COVID-19 Research Database (WHO), and the WHO International Clinical Trials Registry Platform. The search terms contained the target disease group and intervention groups, such as liver neoplasms, CIRT, SARS-CoV-2, and COVID-19. No restrictions were set for the study language, publication date, and publication status. All relevant clinical trials were collected to evaluate CIRT for patients with liver cancer.

All records were imported into the EndNote software of the X9 version (Clarivate Analytics, Clarivate, London, England) for further management and screening. Studies were selected according to the inclusion and exclusion criteria. The articles were reviewed by the researchers independently in two stages for the study screening: the first stage was an evaluation of the titles and abstracts, followed by a full-text review as the second stage. The researchers discussed the discrepancies and re-evaluated the articles until a consensus was reached.

### Data Extraction and Data Analysis

Data were extracted from each included article using standardized forms. The subset of interventions that satisfied the inclusion criteria was kept in the analysis after having discarded the groups that did not satisfy the inclusion criteria when the trials have multiple groups. The list of the collected data included: (1) the basic characteristics of the included studies; (2) the outcomes from the research results. All data were extracted from the text, tables, or figures of the included papers. CIRT was assessed using the method of qualitative synthetic analyses due to its development stage. The data of CIRT were synthesized in both tabular and narrative formats according to the qualitative analysis method of the systematic review.

### Chinese Experience of Fighting Against SARS-CoV-2 and Liver Cancer

The experiences of fighting against SARS-CoV-2 from different countries are necessary due to the unprecedented crisis worldwide. We explored the optimal strategy for treating ULC in the context of the COVID-19 crisis *via* the included studies combined with the experience of Chinese citizens. No ethical approval or patient consent was required for the systematic review as the data originated from previously published studies online. The clinical trial of the first Chinese carbon ion therapy system (CITS) in Wuwei, China was conducted in accordance with the Good Clinical Practice Guidelines and the Declaration of Helsinki, and this trial was approved by the ethics committee of the research institute. All patients provided written informed consent.

## Results

### Results of Study Search and Screening

**Figure 1:**
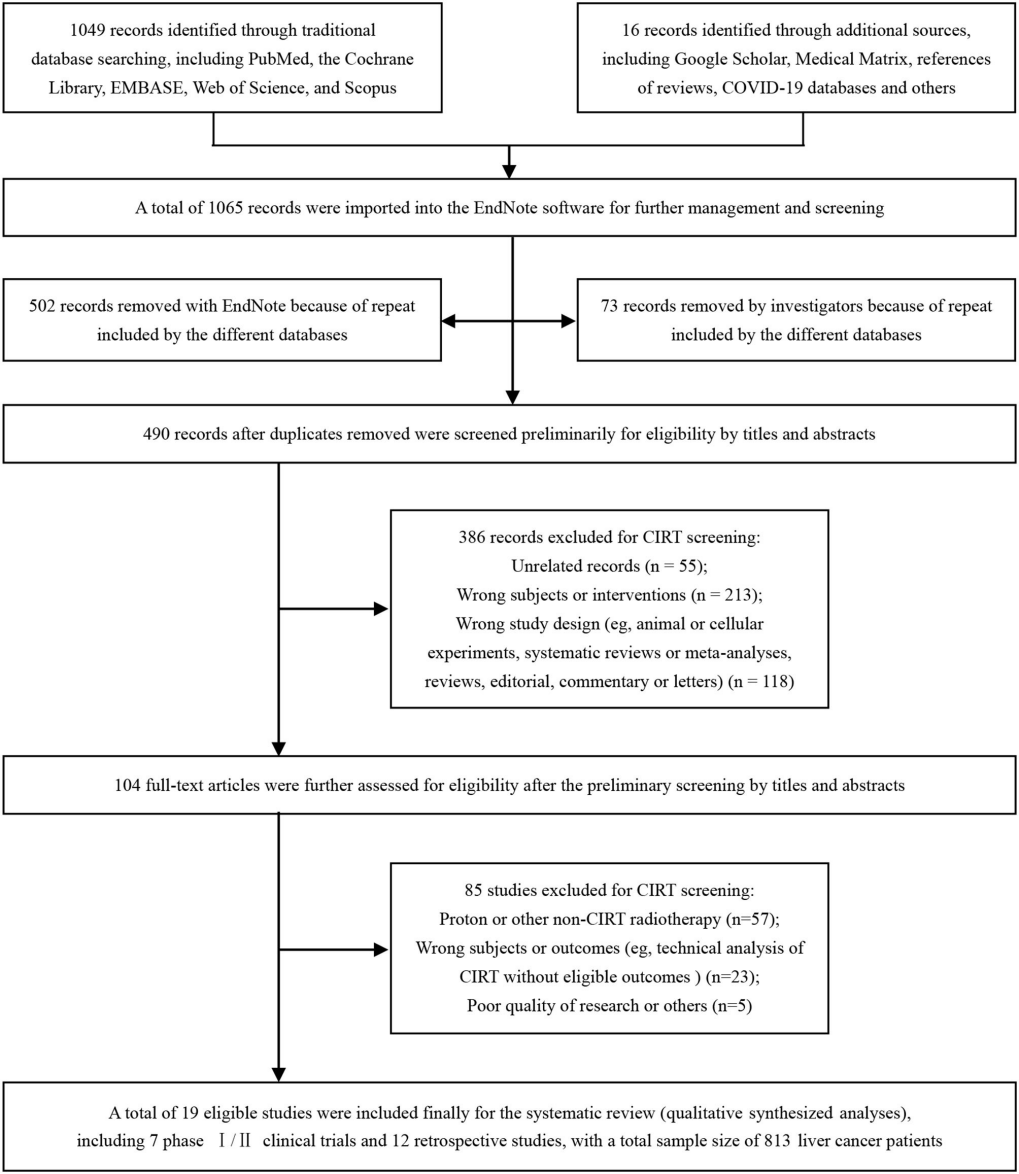
Identification flow chart of the studies to evaluate carbon ion radiotherapy (CIRT) for liver cancer. CIRT, carbon ion radiotherapy.

From our systematized search, a total of 1,065 records were imported into the EndNote software for further identification, including 1,049 records identified through traditional database searching and 16 records identified through additional sources. A total of 575 reduplicative records were removed because of the repeats included by the different databases. After the elimination of duplicates, 490 records were screened for eligibility by their titles and abstracts at the first stage and by full-text screening at the second stage. A total of 19 eligible studies (36–54) were finally included (Figure 1).

### Characteristics of the Included Studies

All of the included studies (36–54) were published in Science Citation Index (SCI) journals and included in the Web of Science with good quality reports. The main features of the 19 included studies (36–54) are presented in Table 1. One propensity-score matching study (43) compared CIRT with transarterial chemoembolization (TACE) for liver cancer, while the other studies (36–42, 44–54) have a single-arm design for the evaluation of CIRT with measurements of safety and efficacy. There were seven phase I/II clinical trials (41, 42, 48, 50, 52–54) and 12 retrospective studies (36–40, 43–47, 49, 51). One study (50) was done in Germany, while the other studies all came from Japan (36–49, 51–54). The total sample size was 813 patients at a rough estimate, which contained 807 patients from Japan. We could not calculate the total sample size accurately because of the existence of overlapping populations, however, the bias of data analysis was low risk because CIRT was assessed using the method of qualitative analyses instead of meta-analyses (Table 1).

**Table 1:** Assessment of the basic characteristics of the 19 included studies.

**References**	**Treatment**	**Nation**	**Study design**	**Research year range**	**Cases (*n*)**	**Age (years)**	**M/F (*n*)**	**Child-Pugh A/B/C (*n*)**	**Diameter (cm)**
Shiba et al. (36)	CIRT	Japan	SRS	2013.10–2020.3	11	65^Δ^ (47–76)^#^	8/3	Unclear	3.1^Δ^ (1.5–6.5)^#^
Okazaki et al. (37)	CIRT	Japan	SRS	2011.1–2018.12	9	80^Δ^ (56–85)^#^	7/2	6/3/0	3.4^Δ^ (1.0–4.7)^#^
Takakusagi et al. (38)	CIRT	Japan	CR	Unclear	2	Case 1: 75; Case 2: 76	Case 1: male; Case 2: male	Case 1: A; Case 2: A	Case 1: 1.3; Case 2: 2.9
Shiba et al. (39)	CIRT	Japan	SRS	2011.7–2018.8	11	76^Δ^ (57–86)^#^	9/2	10/1/0	5.3^Δ^ (2.7–11.9)^#^
Yasuda et al. (40)	CIRT	Japan	SRS	2008.12–2013.3	57	75^Δ^ (49–89)^#^	33/24	51/6/0	3.3^Δ^ (1.3–9.5)^#^
Makishima et al. (41)	CIRT	Japan	CTI	Unclear	29	69^Δ^ (46–84)^#^	20/9	Unclear	2.5^Δ^ (1.2–10.2)^#^
Shibuya et al. (42)	CIRT	Japan	CTI	2012.10–2016.4	21	7^§^(<70)^#^, 14^§^(≥70)^#^	14/7	21/0/0	4.8^※^ (3.0–7.8)^#^; 11^§^(<5)^#^, 10^§^(≥5)^#^
Shiba et al. (43)	CIRT vs. TACE	Japan	PSMS	2007.4–2016.9	Total: 34; CIRT:17, TACE:17	CIRT: 75^Δ^ (45–85)^#^; TACE: 78^Δ^ (59–90)^#^	CIRT: 8/9; TACE: 9/8	CIRT: 15/2/0; TACE: 14/3/0	CIRT: 3.0^Δ^ (1.1–6.4)^#^; TACE: 3.0^Δ^ (0.8–6.0)^#^
Shibuya et al. (44)	CIRT	Japan	SRS, MS	2005.4–2014.11	174	73^Δ^ (37–95)^#^, 67^§^(<70)^#^, 107^§^(≥70)^#^	114/60	153/20/0	3.0^Δ^ (0.8-10.3)^#^; 84^§^(<3)^#^, 90^§^(≥3)^#^
Shiba et al. (45)	CIRT	Japan	SRS	2010.9–2016.12	68	Sarcopenia: 77^Δ^ (57–95)^#^; Non-sarcopenia: 74^Δ^ (45–90)^#^	41/27	57/11/0	Sarcopenia: 3.3^Δ^ (1.2–9.0)^#^; Non–sarcopenia: 3.6^Δ^ (0.9–7.7)^#^
Toyama et al. (46)	CIRT	Japan	CR	2014.9–2016.2	1	50	Female	A	5 cm
Shiba et al. (47)	CIRT	Japan	SRS	2011.3–2015.11	31	≥80^#^	22/9	27/4/0	4.5^Δ^ (1.5–9.3)^#^
Kasuya et al. (48)	CIRT	Japan	CTI, CTII	1997–2003	126	68^Δ^ (37–84)^#^	90/36	97/29/0	4.0^Δ^ (1.0–12.0)^#^; 39^§^(≤ 3.0)^#^, 56^§^(>3.0, ≤ 5.0)^#^, 38^§^(>5.0)^#^
Harada et al. (49)	CIRT	Japan	CR	Unclear	1	54	Female	Unclear	6 cm
Habermehl et al. (50)	CIRT	Germany	CTI	Unclear	6	69^Δ^ (53–78)^#^; 3^§^(<70)^#^, 3^§^(≥70)^#^	3/3	4/1/0	3.5^Δ^ (0.9 – 4.5)^#^
Komatsu et al. (51)	CIRT	Japan	SRS	2001.5–2009.1	101	55^§^(<70)^#^, 46^§^(≥70)^#^	73/28	78/20/3	81^§^(<5.0)^#^, 22^§^(5.0–10.0)^#^, 5^§^(>10.0)^#^
Imada et al. (52)	CIRT	Japan	CTI, CTII	2000.4–2003.3	64	69^Δ^ (37–84)^#^	48/16	49/15/0	4.0^Δ^ (1.2–12.0)^#^
Imada et al. (53)	CIRT	Japan	CTI, CTII	1995.4–2000.3	43	66^Δ^ (45–83)^#^	29/14	35/8/0	Unclear
Kato et al. (54)	CIRT	Japan	CTI, CTII	1995.6–1997.2	24	64^Δ^ (54–77)^#^; 4^§^(54–60)^#^, 15^§^(61–70)^#^, 5^§^(71–77)^#^	13/11	16/8/0	5.0^Δ^(2.1–8.5)^#^; 5^§^(≤ 3.0)^#^, 9^§^ (>3.0, ≤ 5.0)^#^, 10^§^(>5.0)^#^

*CIRT, carbon ion radiotherapy; TACE, transarterial chemoembolization; PSMS, propensity-score matching study; CTI, clinical trial, Phase I; CTII, clinical trial, phase II; SRS, single-arm retrospective study; MS, multicenter study; CR, case report; M, male; F, female;*

Δ *median;*

# *range;*

※ *average;*

§ *number of people*.

### Qualitative Synthetic Analysis for CIRT

A total of 19 studies (36–54) were eligible for the qualitative synthetic analysis of CIRT for liver cancer. The main clinical outcome data after CIRT have been summarized in Table 2. Both prospective and retrospective studies from Japan and Germany have demonstrated encouragingly high rates of OS and local control and low rates of hepatotoxicity with CIRT for treating patients with liver cancer. The reported actuarial OS rates ranged from 90 to 100% at 1 year, from 50 to 88% at 3 years, and from 22 to 48.9% at 5 years, respectively. The local control rates ranged from 81 to 93% at 5 years. A total of four patients with grade 3 adverse events of the hepatotoxicity of transaminase level elevation were reported among the 813 patients included in this qualitative analysis. All studies (36–54) affirmed that severe radiation morbidities were uncommon, and no treatment-related deaths of CIRT were observed (Table 2).

**Table 2:** Clinical outcomes of the included CIRT studies for patients with liver cancer.

**References**	**Dose/# Fx/BED10**	**OS**	**LC**	**RILD Definition**	**RILD Rate**	**RILD Deaths**
Shiba et al. (36)	60.0 GyE/4/150 GyE; 60.0 GyE/12/90 GyE 64.8 GyE/12/99.79 GyE	2-year 100%	2-year 61%	CTCAEv4.0; CP Class progression	0%	0%
Okazaki et al. (37)	52.8 GyE/4/122.5 GyE; 52.8 GyE/12/76.03 GyE; 60 GyE/4/150 GyE 60 GyE/12/90 GyE	MST 18.3 months	1-year 100%	Change in CP score	Acute phase CP+1: 44% Late phase CP+1: 33% CP+2: 11%	0%
Takakusagi et al. (38)	48 GyE/2/163.2 GyE; 60 GyE/4/150 GyE	1-year 100%	1-year 100%	CP Class progression	0%	0%
Shiba et al. (39)	52.8 GyE/4/122.5 GyE; 60 GyE/4/150 GyE 60 GyE/12/90 GyE	3-year 64%	3-year 78%	CP Class progression	3 months CP-A → B 18% 6 months CP-A → B 30%	0%
Yasuda et al. (40)	45 GyE/2/146.25 GyE	1-year 97% 3-year 67% 5-year 45%	1-year 98% 3-year 91% 5-year 91%	CTCAEv4.0; Change in CP score	≥G3: 0%; ≥CP+2: 0%	0%
Makishima et al. (41)	36–58 GyE/1/165.6–394.4 GyE	3-year 78%	3-year 82%, high doses; 3-year 28%, lower doses	NCI-CTC/RTOG-ARMSS/EORTC-LRMSS	Acute toxicities G1: 17% G2: 3% Late toxicities G1: 21% G3^§^: 7%	0%
Shibuya et al. (42)	60 GyE/4/150 GyE	1-year 100% 2-year 92.3%	1-year 90.5% 2-year 80.0%	CTCAEv4.0: GGT, AST	Within 90 days ≤ G1: 86% G2: 14% After 90 days ≤ G1: 90% G2: 10%	0%
Shiba et al. (43)	52.8 GyE /4/122.5 GyE; 60 GyE /4/150 GyE; 60 GyE /12/90 GyE	3-year 88%	3-year 80%	CP Class progression	0%	0%
Shibuya et al. (44)	48.0 GyE /2/163.2 GyE; 52.8–60.0 GyE /4/122.5–150 GyE	1-year 95.4% 2-year 82.5% 3-year 73.3%	1-year 94.6% 2-year 87.7% 3-year 81.0%	CTCAEv4.0: AST, ALT	1.7%; one case with G3 ALT elevation	0%
Shiba et al. (45)	52.8, 60 GyE/4/122.5, 150 GyE	Sarcopenia: 3-year 66% Non-sarcopenia: 3-year 77%	Sarcopenia: 3-year 81% Non-sarcopenia: 3-year 72%	CTCAEv4.0: AST, ALT	Acute toxicities G1: 7% G2: 3% Late toxicities G1: 4% G2: 4%	0%
Toyama et al. (46)	60 GyE/4/150 GyE	1-year 100%	1-year 100%	NR	0%	0%
Shiba et al. (47)	Close-GI-tract: 60 GyE/12/90 GyE Others: 52.8–60 GyE/4/122.5–150 GyE	2-year 82%	2-year 89%	CP score and Class progression	3 months CP+1: 13% CP+2: 3% 6 months CP+1: 16% CP+2: 3% CP-A → B 3%	0%
Kasuya et al. (48)	Phase I: 54, 48, 48 GyE/12, 8, 4/78.3, 76.8 GyE/105.6 GyE Phase II: 52.8 GyE/4/122.5 GyE	1-year 90% 3-year 50% 5-year 25%	1-year 95% 3-year 91% 5-year 90%	CP score and Class progression	3 months CP+1: 29% CP+2: 3% CP+3: 1% 6 months CP+1: 22% CP+2: 5% CP-A → B 13%	0%
Harada et al. (49)	36 GyE/1/165.6 GyE	8-year 100%	8-year 100%	NR	0%	0%
Habermehl et al. (50)	40 GyE/4/80 GyE	MST 11 months	Crude 100%	CTCAEv4.03: AST, ALT	≥G2: 40%	0%
Komatsu et al. (51)	52.8–76.0 GyE/4–20/87.6–122.5 GyE	5-year 36%	5-year 93%	CTCAEv2: AST, ALT	≥G2: 3% G3: 1%	0%
Imada et al. (52)	52.8 GyE/4/122.5 GyE	5-year 22%	5-year 88%	Change in CP score	CP+1: 84% CP+2: 16%	0%
Imada et al. (53)	48.0–79.5 GyE/4–15/65.8–122.5 GyE	Larger enlargement group 3-year 80.0% 5-year 48.9% Smaller enlargement group 3-year 52.2% 5-year 29.4%	NR	NR	NR	0%
Kato et al. (54)	49.5–79.5 GyE/15/65.8–121.6 GyE	1-year 92% 3-year 50% 5-year 25%	1-year 92% 3-year 81% 5-year 81%	Change in CP score	CP+1: 30% CP+2: 22%	0%

*Fx, fraction; BED10, biologic equivalent dose with α/β of 10; OS, overall survival; LC, local control; RILD, radiation-induced liver disease; NR, not reported; CP, Child-Pugh score; MST, median survival time; GGT, Gamma-glutamyltransferase; AST, Aspartate aminotransferase; ALT, Alanine aminotransferase; NCI-CTC, National Cancer Institute – Common Toxicity Criteria; RTOG-ARMSS, Radiation Therapy Oncology Group, Acute Radiation Morbidity Scoring System; EORTC-LRMSS, European Organization for Research and Treatment of Cancer, Late Radiation Morbidity Scoring System; CTCAE, Common Terminology Criteria for Adverse Events;*

§ *2 temporary grade 3 liver toxicity cases due to biliary obstruction were observed at 9 and 21 months after the treatment as late toxicity at 53 Gy (RBE), but both fully recovered*.

Shiba et al. (43) reported a propensity-score matching (PSM) study that compared CIRT with TACE for patients with single hepatocellular carcinoma. Seventeen matched pairs of patients from each group were included for further analyses after PSM. The results demonstrated that CIRT significantly improved the clinical outcomes over TACE with regard to the 3-year OS rate (88% with CIRT vs. 58% with TACE, *p* < 0.05), 3-year local control rate (80% with CIRT vs. 26% with TACE, *p* < 0.01), and 3-year progression-free survival rate (51% with CIRT vs. 15% with TACE, *p* < 0.05), respectively. Compared with TACE, CIRT was associated with a significant reduction regarding the number of patients whose liver function progressed to a worse Child-Pugh class within 3 months from the initiation of treatment (*p* < 0.01). There were two studies (41, 49) regarding single fraction CIRT for metastatic liver cancer, and the results showed that single fraction CIRT was safe and effective. As a special case, a woman with a 6 cm chemo-resistant metastatic liver tumor from breast cancer was successfully cured with a single shot of 36-GyE CIRT, and the woman survived more than 8 years without local recurrence (49).

### Chinese Experience of Fighting Against SARS-CoV-2 and Liver Cancer

The CITS in Wuwei, China, which was independently developed by the Institute of Modern Physics (IMP), Chinese Academy of Sciences in 1993, successfully completed the treatment of 46 cancerous cases as a clinical trial and was officially registered as a medical device of Class 3 in China on September 29, 2019. The CITS in Wuwei is the first Chinese CIRT equipment with the serial number 20193050713 and the type specification HIMM-01-GS-WW-1, and several CITSs in other areas of China are now under construction. A total of 47 cancer cases were enrolled into the clinical trial for the medical device registration of CITS in Wuwei. One patient withdrew from the trial after enrolment, and 46 subjects completed the trial finally. There were a total of seven patients with ULC in the trial, including six cases with primary hepatocellular carcinoma and one case with hepatic metastasis from rectal cancer. All of these patients were advanced and intractable cancer cases. No severe radiation morbidities and treatment-related deaths of CIRT were observed during the treatment and follow-up. The data acquired from the clinical trial in Wuwei, China demonstrated that the Chinese CITS encouraged safety and anti-cancer effectiveness in treating liver cancer (Table 3).

**Table 3:** Clinical outcomes of CIRT with the first Chinese carbon ion therapy system (CITS) for patients with liver cancer.

**Items classification**	**Case 1**	**Case 2**	**Case 3**	**Case 4**	**Case 5**	**Case 6**	**Case 7**
Age (years)	72	49	63	68	49	44	72
Gender	Male	Male	Male	Male	Male	Male	Female
Pathological type	Primary HCC	Primary HCC	Primary HCC	Primary HCC	Primary HCC	Primary HCC	Hepatic metastasis of rectal cancer
Treatment-related deaths	No	No	No	No	No	No	No
Severe radiation morbidities	No	No	No	No	No	No	No
Efficacy at 3 mon	PR	SD	SD	SD	PR	PR	SD
Efficacy at 6 mon	PR	PR	SD	SD	SD	CR	SD
Survival at 0.5 year	Yes	Yes	Yes	Yes	Yes	Yes	Yes
Survival at 1 year	Yes	Yes	Yes	Yes	Yes	Yes	Yes

*HCC, hepatocellular carcinoma; mon, months post-treatment; CR, complete response; PR, partial response; SD, stable disease; CIRT, carbon ion radiotherapy; CITS, carbon ion therapy system; ULC, unresectable liver cancer*.

**Figure 2:**
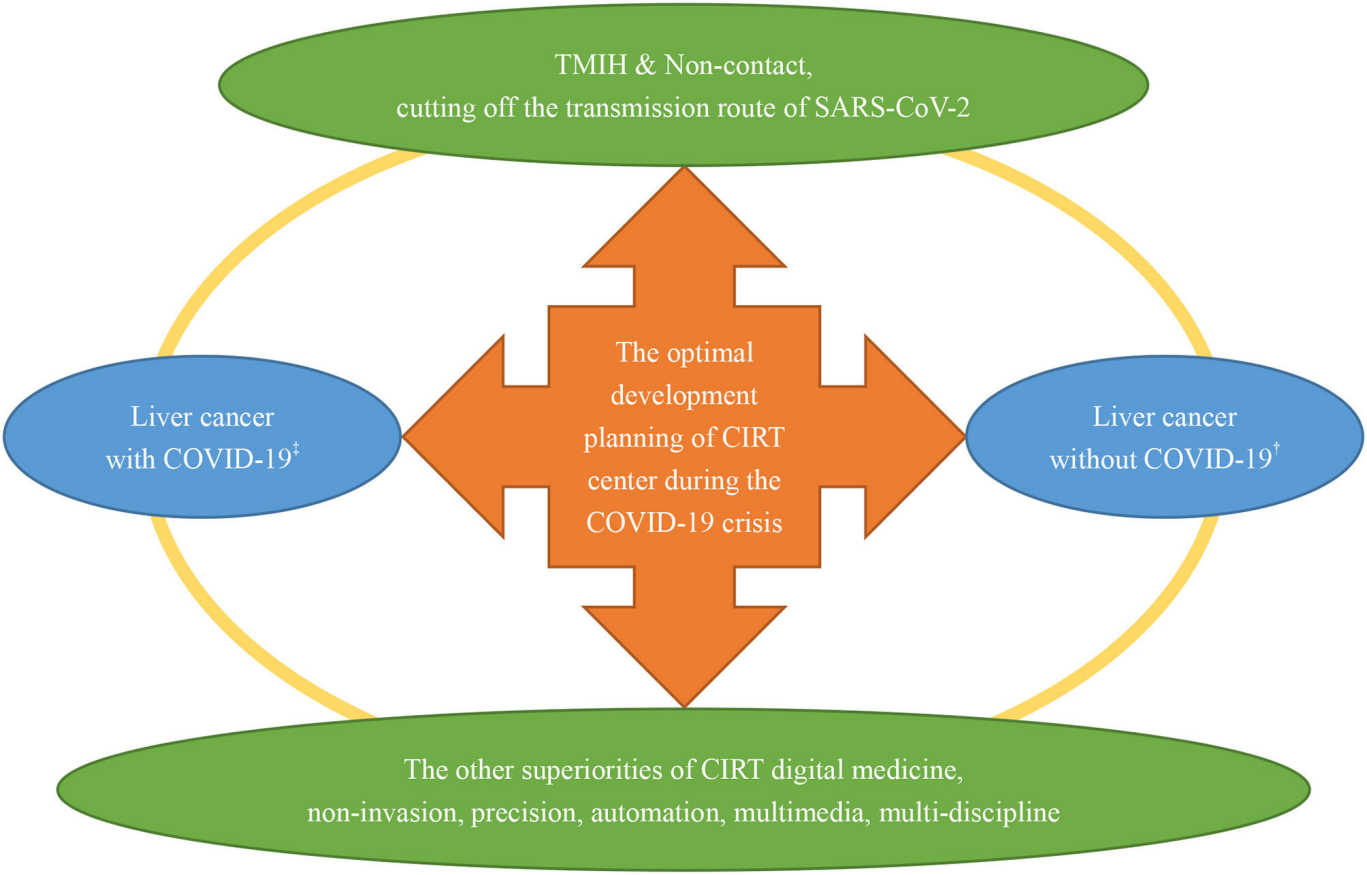
A schematic diagram for the development planning of the CIRT center during the COVID-19 crisis. CIRT, carbon ion radiotherapy; ^‡^liver cancer patients with concurrent SARS-CoV-2 infection; liver cancer patients without SARS-CoV-2 infection.

According to the experience of Chinese citizens, compared with other locoregional treatment (LRT) (including surgical resection, thermal ablation, transarterial chemoembolization, percutaneous ethanol injection, and so on), CIRT allows TMIH with controllable risk of SARS-CoV-2 infection in the whole process of treatment for ULC. Compared with photon (or proton) radiotherapy modalities, CIRT could achieve the optimal utilization rate of hospitalization and outpatient care (UHO). Therefore, non-invasive CIRT is identified as the optimal treatment strategy for appropriate patients with ULC concerning the need to cut off the transmission route of SARS-CoV-2 and to improve the capacity of healthcare service in the context of the unprecedented COVID-19 crisis. Based on Chinese foundations, ultramodern projects of CIRT have been planning and preparing to bring its superiorities into full play. A schematic diagram for the development planning of the CIRT center is exhibited in Figure 2. As shown in Figure 2, the new-style CIRT center has the excellent ability of TMIH and is non-contact. In addition, the burgeoning digital medicine of CIRT possesses many other superiorities including non-invasion, precision, automation, multimedia, and multi-discipline, which is beneficial to protect vulnerable cancer groups from SARS-CoV-2 infection by minimizing toxicities to cancer patients (especially for immune-system). Therefore, the Chinese CITSs will play a crucial role in pulling the appropriate patients with liver cancer through crises such as the COVID-19 pandemic.

## Discussion

Globally, COVID-19 has caused unprecedented social turmoil, triggering a comprehensive transformation of global healthcare systems (3–6, 13, 55–59). From the perspective of cancer patients, any policy or strategy that neglects their benefit due to the COVID-19 pandemic, such as delaying treatment in some guidelines after the COVID-19 outbreak as a typical example, has immensely increased the risk of cancerous malignant death (13, 60–62). Based on fully respecting the interests of patients with ULC worldwide, herein, we put forward a kind of brand new perspective and method to fight against SARS-CoV-2 and ULC simultaneously by optimizing the treatment strategy of ULC.

### CIRT for Liver Cancer During the COVID-19 Crisis

Aitken et al. (32) suggested that photon-based stereotactic ablative radiotherapy (SABR) could be considered as an effective and feasible alternative to surgery for patients with liver cancer because of the unprecedented impacts of the COVID-19 pandemic on the United Kingdom cancer services. Maybe the COVID-19 pandemic is primetime for the application of SABR in cancer treatment and the single fraction SABR has been further placed great expectations (31, 34). Why was CIRT identified as the optimal strategy for ULC in the context of the COVID-19 crisis? Primarily, TMIH and non-invasiveness are the crucial considerations for decision-making (7–12). What is more, CIRT possesses multidimensional superiorities compared with either photon or proton radiotherapy (21, 29, 63, 64). CIRT is superior to SABR for treating patients with liver cancer due to its unique advantages in terms of target conformity and normal liver tissue sparing, relative biological effectiveness (RBE), duration of treatment, risk of subsequent primary cancers, and so on (20, 21, 29, 30, 35, 63–68). Additionally, CIRT is beneficial to protect the immune system and activate specific anti-cancer immunity by triggering the immunoreaction on account of its excellent superiorities in the aspects of dose localization and RBE (21–28), which is extremely significant for patients to fight against cancer and SARS-CoV-2 synchronously (7–12).

The qualitative synthetic analyses results of CIRT for liver cancer demonstrated encouragingly high rates of OS and local control and low rates of hepatotoxicity. One of the most limiting factors of the use of radiotherapy for liver cancer is the significantly poor radiation tolerance of the normal liver tissues, especially when the liver function is impaired by some chronic liver disease (50, 51). CIRT is the optimal radiation modality for maximizing anti-cancer effectiveness while minimizing radiation-induced hepatotoxicity due to its inherently physical and biological superiorities (65, 66). A propensity-score matching (PSM) study (43), a method that could minimize potential selection bias of patients in retrospective studies by mimicking some characteristics of RCT, has manifested that CIRT possesses significantly more effectiveness and less toxicity than TACE in the treatment of liver cancer. A synthetical study by Zhang et al. indicated that CIRT is more therapeutically beneficial with adequate safety than the radiotherapy modality of proton or photon (20). Based on the evidence, CIRT was identified as the optimal strategy for appropriate patients with ULC during the COVID-19 crisis, especially the single fraction CIRT for specific ULC patients concerning the need to minimize the risk of SARS-CoV-2 infection.

### China's Experience in Combating COVID-19 and Liver Cancer

The statistics showed that liver cancer was the third leading cause of cancer death worldwide in 2020, with about 906,000 new cases and 830,000 deaths annually (69). In addition, nearly half of the world's morbidity and mortality of liver cancers are distributed in China (69, 70). There exists a dilemmatic predicament with regards to conventional therapies for liver cancer in the context of the COVID-19 crisis, which is significantly different from the real world before the COVID-19 pandemic (7–12). Protecting patients from the SARS-CoV-2 infection generally results in delaying (even giving up) conventional treatment for liver cancer patients on account of the high risk of SARS-CoV-2 infection caused by conventional treatment (7–13). But on the other hand, what is the optimal alternative strategy for the conventional treatment for reducing the risk of cancer malignant death? In order to find the way out of this unprecedented predicament, we have identified CIRT as the optimal treatment strategy for applicative patients with ULC after a comprehensive investigation.

The IMP of China has started to develop CITS independently since 1993, and now, we have many original innovations not only in the equipment and clinical technology but also in the supporting theoretical basis, such as the relative biological effectiveness (RBE) modeling for CIRT (71). Why was CIRT identified as the optimal strategy for ULC concerning the need to minimize the risk of SARS-CoV-2 infection? Based on the Chinese CITS foundations and the successful experiences in fighting COVID-19, the reasons could be summarized as follows. (1) The first reason is with respect to cutting off transmission routes. CIRT has the excellent ability of TMIH and non-contact in the whole process of treatment for ULC, thereby allowing quarantine and keeping a sufficient person-to-person physical distance between patients and others. This peculiarity of CIRT is crucial to realize cutting off the transmission route of SARS-CoV-2. (2) The second reason is with respect to protecting vulnerable populations. (a) CIRT is a non-invasive and precision treatment modality for ULC. Therefore, CIRT could minimize toxicities to patients (especially for the immune system) and concurrently obtain excellent anti-cancer effectiveness, which is significant to preserve patients with ULC in a relatively good systemic and immune condition for fighting against SARS-CoV-2 and cancer in the context of the COVID-19 crisis. (b) Compared with photon (or proton) radiotherapy modalities, CIRT is associated with significantly fewer fractions and a shorter duration of hospitalization, which is beneficial to reduce the risk of nosocomial cross-infection of SARS-CoV-2, as well as to increase the turnover rate of hospitalization. Accelerating the turnover rate of hospitalization is necessary for healthcare systems in the context of the COVID-19 crisis because of the widespread shortage of medical resources. (c) All the advantages of CIRT, especially the unique capacity of a single fraction regimen for completing the treatment, make it feasible to offer an outpatient ablative approach with minimal hospital footfall and duration, which is significant to minimize the risk of SARS-CoV-2 infection by minimizing the exposure frequency of nosocomial SARS-CoV-2 sources. Therefore, single fraction CIRT would be the optimal choice of radiotherapy during the COVID-19 crisis for specific patients with liver cancer. As a response to this pandemic, the use of CIRT will become more and more important due to the increasing need to offer optimal risk-benefit results. We propose that personalized treatment recommendations should be addressed to minimize the risk of SARS-CoV-2 infection and malignant death synchronously along with meticulous personal protective protocols for liver cancer patients.

In the summer of 2021, the SARS-CoV-2 Delta Variant surge has caused a new wave of the epidemic peak in America and other countries (4, 59, 72, 73). As a matter of fact, there has been an unprecedented shortage of hospital beds and other medical resources due to the severe COVID-19 epidemic, causing the increasing death of both patients with and without COVID-19 (4, 5, 16, 59). Therefore, it is imperative to accelerate the turnover rate of hospitalization and increase the capacity of outpatient care in the context of the COVID-19 crisis (5, 16, 32, 59). CIRT could maximize the UHO of ULC patients on account of the shortened hospital stay (due to shortened treatment course) and the excellent capacity of the outpatient approach. Consequently, CIRT is helpful not only to improve the capacity of medical service but also to minimize the risk of nosocomial cross-infection of SARS-CoV-2 by reducing the exposure frequency and total duration in the SARS-CoV-2 environment.

### Study Limitations

The present study has a few limitations. With the exception of a PSM study (43) for CIRT in comparison with TACE, the other studies (36–42, 44–54) for the CIRT assessment were all case reports or single-arm studies lacking a contrastive control group. This is mainly due to the growing stage of CIRT. While the evidential strength of the CIRT assessment is limited, the evidence of CIRT is urgently needed and important for global oncologists to fight against SARS-CoV-2 and liver cancer concurrently in the context of the COVID-19 pandemic because of its unique superiorities. All of this evidence and experiences are necessary for decision-making because timely life-saving is the foremost principle in the unprecedented crisis worldwide.

## Conclusions and Future Prospects

In order to optimize the treatment strategy of patients with ULC due to the COVID-19 crisis worldwide, multi-angle methods were implemented to evaluate the non-invasive CIRT for treating ULC, from which we concluded that CIRT could obtain favorable anti-cancer effectiveness and concurrently, minimize toxicities to patients for preserving patients in a relatively good systemic and immune condition. In addition, CIRT has the ability of TMIH with the controllable risk of SARS-CoV-2 infection exposure, as well as the optimal utilization rate of both hospitalization and outpatient care concurrently. Therefore, we have definitively judged CIRT as the optimal treatment strategy for appropriate patients with ULC when we need to minimize the risk of SARS-CoV-2 infection and improve the capacity of medical service in the COVID-19 crisis. We believe that CIRT will be greatly helpful to reduce the risk of SARS-CoV-2 infection and cancer malignant death concurrently during the COVID-19 pandemic. We also firmly believe that the trajectory of this unprecedented pandemic caused by SARS-CoV-2 will become better and better worldwide with international cooperation and mutual assistance, innovation, and sharing.

## Data Availability Statement

The original contributions presented in the study are included in the article/supplementary material, further inquiries can be directed to the corresponding author.

## Ethics Statement

The studies involving human participants were reviewed and approved by the Ethics Committee of Gansu Provincial Cancer Hospital and the Ethics Committee of Gansu Wuwei Tumor Hospital. The patients/participants provided their written informed consent to participate in this study.

## Author Contributions

ZL and QL developed the study conception and design. ZL, QL, and XW supervised the whole study process and coordinated all the work. ZL, QL, XW, SL, and WC collected the data and prepared the figures and tables. ZL, QL, XJ, and XGL contributed to selecting the analytical tools and methods. ZL, QL, ZD, XXL, XZ, PL, HZ, QZ, HL, and RL analyzed, interpreted, reviewed, and cross-checked the data. ZL and QL wrote and revised the manuscript. All authors critically reviewed and approved the final manuscript.

## Funding

This work was jointly supported by the China Postdoctoral Science Foundation (Grant No. 2019M663860), the National Natural Science Foundation of China (Grant No. 11875299), the Key Deployment Project of the Chinese Academy of Sciences (Grant No. KFZD-SW-222), and the West Light Foundation of Chinese Academy of Sciences (Grant No. 29Y86205).

## Conflict of Interest

The authors declare that the research was conducted in the absence of any commercial or financial relationships that could be construed as a potential conflict of interest.

## Publisher's Note

All claims expressed in this article are solely those of the authors and do not necessarily represent those of their affiliated organizations, or those of the publisher, the editors and the reviewers. Any product that may be evaluated in this article, or claim that may be made by its manufacturer, is not guaranteed or endorsed by the publisher.
